# Barriers and facilitators for coherent rehabilitation among people with inflammatory arthritis – a qualitative interview study

**DOI:** 10.1186/s12913-022-08773-x

**Published:** 2022-11-14

**Authors:** Helle Feddersen, Jens Søndergaard, Lena Andersen, Bettina Munksgaard, Jette Primdahl

**Affiliations:** 1 University College of Southern Denmark, Campusalle 20, 6200 Aabenraa, Denmark; 2 Danish Hospital for Rheumatic Diseases, University Hospital of Southern Denmark, Sønderborg, Denmark; 3 Department of Regional Health Research, University of Southern Denmark, Odense, Denmark; 4 The Research Unit for General Practice, Department of Public Health, University of Southern Denmark, Odense, Denmark; 5 Sygehus Sønderjylland, University Hospital of Southern Denmark, Aabenraa, Denmark

**Keywords:** Person-centred care, Integrated care, Complex adaptive systems, Inpatient rehabilitation, Chronic disease, Empowerment, Case-manager, Navigator

## Abstract

**Background:**

People with chronic diseases have contact with several different professionals across hospital wards, municipality services and general practice and often experience lack of coherence. The purpose was to explore perceived barriers and facilitators to coherent rehabilitation pathways for health care users with inflammatory arthritis and how coherence can be improved.

**Methods:**

Semi-structured individual interviews were conducted before a planned inpatient rehabilitation stay, 2-3 weeks and 4-6 months after discharge. Thematic reflexive analysis guided the analysis of data. Concepts of person-centred care, complex adaptive systems and integrated care were applied in the interpretations.

**Results:**

In all, 11 participants with IA were included. There was one overarching theme, The importance of a person-centred approach, illuminating the significance of professionals who respect healthcare user’ preferences. To use a person-centred approach, demands professionals who are interested in exploring the persons own values, preferences and experiences and incorporate these when planning care and rehabilitation*.*Connected to the overarching theme, three sub-themes were derived; 1) Experiences of empowerment and dis-empowerment, covering that most want to be in control and act themselves, but felt overwhelmed and lost energy and they tended to give up; 2) Experiences of communication and coordination, encompass how people feel forced to take on coordination and communication tasks themselves although they do not always feel qualified for this. Some asked for a coordination person and 3) Facing everyday life after discharge, covering how initiatives taken by professionals were not always experienced as helpful after discharge. Some gave up and some tried to find alternative paths themselves.

**Conclusion:**

Professionals taking a person-centred approach facilitated coherent rehabilitation pathways. This encompassed care with respect for individual needs and professionals who empowered patients to self-management. Furthermore, to be aware that interprofessional communication and coordination need to take place both between professional within the same department, between departments and between professionals in different sectors.

After discharge, some patients were challenged in their everyday life when trying to follow the advice from the professionals. Professionals, who do not use a person-centred approach, hinder coherence. Patients thus feel compelled to take on communication and coordination tasks.

## Background

Many healthcare users with chronic diseases do not experience coherence in their rehabilitation pathways. This can be attributed to a highly specialised and complex healthcare system and complex health and social care needs, where provided services are offered across different professional groups, departments, and sectors [1–6]. In this study, rehabilitation pathways denote services, which are organized to maintain or increase healthcare users’ bio-psycho-social functioning as well as increasing active participation in society [7, 8].

Lack of communication and coordination in connection with the delivery of information regarding treatment and care between professionals, e.g. when healthcare users’ are transferred between different hospital wards [6, 9–11] or between primary and secondary health care, may cause fragmentation of rehabilitation pathways [9, 12–14].

These deficiencies in communication and coordination can lead to medication errors and negative consequences in the form of lack of progress and coherence in the clinical pathway [12]. Healthcare users report that they do not always receive the necessary information about care, treatment and rehabilitation and they experience that their pathways are out of control [6].

In addition, organisational factors can constitute a barrier to healthcare users’ experience of coherence [5, 15–19]. Specialisation and complexity in the healthcare system can make it problematic for the professionals to have in-depth knowledge of rehabilitation services across sectors and of other professionals’ competencies, which can challenge coherence in the pathways [20–22]. Thus, healthcare users are left with the responsibility themselves to create coherence between the various rehabilitation services across professional groups and sectors [5, 6, 10, 11, 23].

### The Danish healthcare system

The Danish healthcare system is internationally recognized for being efficient; however Organisation for Economic Co-operation and Development also points out problems with communication across primary and secondary sectors [24]. The Danish healthcare system is primarily public financed and is organised in three administrative levels: a national, regional, and local level. The national level is responsible for the overall healthcare system and the regional level is responsible for healthcare services provided in the hospitals and by General Practitioners (GPs) [25] and constitutes the secondary sector. The local level provides primary prevention, generic rehabilitation services and home care in the municipalities and constitutes the primary sector. Furthermore, the municipalities offer social care [25].

We have chosen healthcare users with chronic Inflammatory arthritis (IA) as the case in this study to explore challenges for coherent rehabilitation pathways, as this group of healthcare users need long-term services that, in addition to medical treatment, also address the physical, psychological and social consequences derived from the disease [26–30]. They may thus need rehabilitation, which involves different professional groups across departments and sectors.

Challenges with fragmentation in the provided healthcare services have traditionally been explored within organisational perspectives and have been directed to professional and clinical perspectives instead of the users’ perspective [31, 32]. The purpose of this study was therefore to explore perceived barriers and facilitators to coherent rehabilitation pathways for healthcare users with inflammatory arthritis and how coherence can be improved.

## Methods

Consolidated criteria for reporting qualitative research have guided reporting of this study [33].

### Design

The study was planned as a qualitative study informed by symbolic interactionism [34], a theoretical approach in which meaning is a core element and thus people act based on the meanings things and other people have for them [34]. This implied that healthcare users with IA were considered to try to make sense of the social interactions between them and the health professionals they met in their rehabilitation pathways, and that they interacted according to their sensemaking of these interactions. To get insight in the target group’s sensemaking of experiences with barriers and facilitators to achieve coherent rehabilitation in their pathways, we found it appropriate to accomplish this study with data from qualitative interviews [35].

### Setting

We recruited participants among healthcare users referred to a specialised rehabilitation stay at the Danish Hospital for Rheumatic Diseases, which is the only Danish hospital offering specialised interdisciplinary inpatient rheumatology rehabilitation. The inpatients are referred by a rheumatologist or their GP. Furthermore, criteria for referral to the specialised inpatient rehabilitation is an assessment that there is a rehabilitation potential, and that the patient has already tried rehabilitation offers in the primary sector.

The rehabilitation stays usually last two weeks and encompass an interdisciplinary team-based intervention encompassing rheumatologists, nurses, nurse assistants, physiotherapists, occupational therapists, a social worker, dietitian and if relevant, a specialist in orthopaedic surgery. Rehabilitation interventions may include group or individual exercise and education. Educational topics can include self-management of pain, fatigue, sleep and living with chronic illness.

### Participants

We used the concept of information power [36] to guide and evaluate the quality of data generation and thereby to guide adequate sample size for the study. Reflections in relation to information power resembles what may be called data saturation in other traditions i.e. grounded theory [37].

Reflections on information power imply to consider the following items: study aim, sample specificity, quality of dialogue, use of established theory, and analysis strategy when including participants. This means that the concrete sample size was not determined in advance. Regarding study aim and sample specificity, we were guided by purposeful sampling to include participants with IA, who had several contacts with professionals across different departments and sectors during their rehabilitation pathways. The latter to ensure diversity in events [36] and ensure rich data to answer the study aim. In addition, we were guided by the concept of maximum variation [38]. We aimed to achieve maximum variation in relation to age, sex, and duration of disease. We aimed to establish a positive and confident relation and achieve a good dialogue [36] during the interviews. During the recruitment process and the initial analysis, the quality of data was evaluated to decide if further data was needed and if so, what kind of data and by whom [36]?

A secretary sent a letter together with the invitation letter for the rehabilitation stay to all patients offered a stay for a period of 2-3 weeks. We did not record the exact number of invitations sent out. The patients who were interested in participating contacted us for further information about the study. During the first telephone contact with potential participants, we assessed whether the inclusion of the participant would add relevant and comprehensive data as they needed to have had several contacts during their rehabilitation pathway and be able to articulate their experiences. In addition, we selected participants to achieve maximum variation as described. Each individual participant also had to be willing to take part in three interviews. Oral and written consent were obtained before inclusion.

### Data generation

**Fig. 1:**
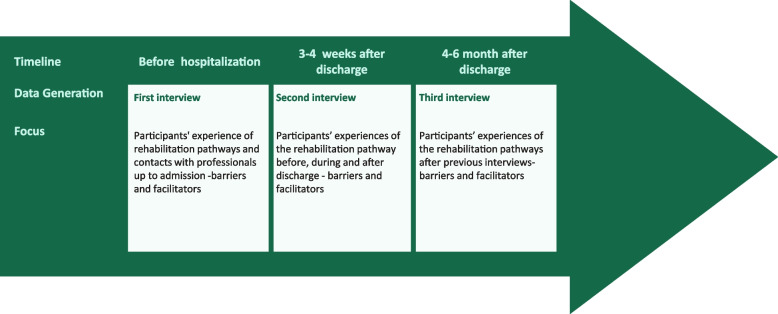
Data generation, illuminates the timeline of data generation, which encompassed semi-structured interviews with the included participants before their rehabilitation stay, 3-4 weeks and 4-6 months after discharge

Data generation encompassed semi-structured interviews [39] with the included participants before their rehabilitation stay, 3-4 weeks and 4-6 months after discharge (Figure 1). Individual semi-structured interviews were chosen as we wanted to explore the experiences of the healthcare users in relation to the specific aim of the study. This inferred a balance between asking structured and open questions and to pursue the participants responses in relation to the aim as well as probing questions to further explore unexpected topics.

The time span from the first to the third interview was approximately 6 months. The interviews focused on the participants’ experiences of which professionals within the healthcare system or the social system they had engaged with and how they experienced the work of these professionals had facilitated or limited coherence in their rehabilitation pathway.

For the initial semi-structured interviews before admission, interview guides were prepared (please see Table 1) based on literature and knowledge from previous research [2, 5, 13] and the two patient research partners’ experiences.

**Table 1:** Interview guides

Interview guide, first interview	
Content	Questions
Experience of pathways and contacts with professionals up to the admission – barrieres and facilitators	• Please, tell about the contacts and experiences you have had with the professionals regarding your illness • Who were involved and what did they do? • What have you done yourself during this process? • Please, tell me about what is important for whether you experience coherence in your pathway or not
Interview guide, second interview	
Content	Questions
Coherence regarding specialised rehabilitation stay	• How did you experience the hospitalization and what did you actually achieve? • How did you experience cooperation and coordination between the professionals? Was there coherence between the different activities/ efforts? • How did the professionals during the hospitalization collaborate with the other professionals about your pathway (e.g. other hospitals, own doctor, physiotherapist, social worker in the municipality)? • What is important for whether you experience coherence in relation to the admission?
Coherence regarding discharge	• Was a decision made on specific follow-up from the admission (what, how, when)? • Were new initiatives launched (eg free physiotherapy)? • Did you experience that the physician and the nurse had knowledge of your pathway and got you "out the door" in a good way?
Coherence regarding the time after rehabilitation stay in the hospital	• How was it to get back home again? • Did you need any follow-up after the admission (if so, who did what)?
Coherence in the rehabilitation pathway from referral during the admission and after discharge	• Do you experience that there has been coherence in your pathway from referral during admission to discharge and the time after discharge? • If yes: what/ who contributed to this coherence? • If no: What/ who was been missing? • Did you have to do anything yourself to create coherence? If so, what?
Interview guide, third interview	
Content	Questions
Coordination between the various rehabilitation services before, during hospitalisation and after discharge – barrieres and facilitators	• Did you experience a need for coordination between different services, e.g. own doctor, hospital or municipality after discharge? • If we look at your pathway up to, during admission and after discharge, how have you experienced coherence and coordination between the professionals you have been in contact with? • What hindered or helped to create coherence and coordination? • Did you have to do anything yourself to create coherence? If so, what?
All interviews	
Content	Question
Introduction	•How are you doing in relation to your illness?
Closing	•Do you have any information about coherence in your pathway, that I did not ask for?
Coherence	•Whey questioning coherence in their pathway –this could be worded as e.g.: what makes you sure or not, that the professionals communicate and coordinate the provided services across different appartments and sectors? What makes you sure or not, that several initiatives (e.g. referrals, tests, treatments) provides continuity in your pathway? Other aspects which make you safe or feeling confortable What makes you feel the opposite?

The second and third interview contained issues discussed in the first interview and the participants’ experiences during and after discharge from the specialised rehabilitation.

The interviews were conducted in the participants’ home or in a location in accordance with the participant’s desire, i.e., one interview took place at the participant’s workplace.

The interviews lasted for 16-111 minutes (in average 52 min). All interviews were digitally recorded and transcribed verbatim [38].

The team behind this article was familiar with the healthcare system. The team consisted of two patient research partners (females), both diagnosed with IA, a professor and GP (male), a registered nurse (RN), professor in rehabilitation (female) and a lecturer, PhD, and RN (female). The latter performed data collection together with a previous employed researcher from our research department. All members of the team participated in planning the study, the analysis and drafting of the article.

### Analysis

Thematic reflexive analysis (TA) [40, 41] guided the analysis and interpretation of data. TA is a theoretical flexible method; however, it is underlined that research can never be conducted in a philosophical or theoretical vacuum [40]. In accordance with our symbolic interactionist approach [34], the meaning the participants ascribed to their experiences of their rehabilitation pathways was essential for the analysis and how we could interpret their stories.

The analysis followed a six-phase process for data engagement, coding and theme development [40]. Five of the six phases are described in Table 2. Phase number 6 is writing the report. Working with the phases is not strictly a linear process but is an iterative and recursive process characterised by moving back and forth between the phases. Before the six-phase process was carried out, condensed case descriptions of the participants were developed to get an overview over every participant’s pathway. We conducted the analysis where two of the researchers read all the transcripts, a few read some and all read the detailed case descriptions. All participated in the discussions during the iterative development of themes where the data behind the codes and themes were discussed.

**Table 2:** Overview over the phases

Phases*	Content	What did we do	When to involve theories in the interpretations
Phase 1	Data familiarisation and writing familiarisation notes	Read transcribed interviews several times	• Inductive, data-driven reading • Open to multiple possible theoretical interpretations
Phase 2	Systematic data coding	Labelling segments of relevant text with relevance to the aim of the study – and collate tagged data.	• Initially open, inductive • Returning to data, modify early codes
Phase 3	Generating initial themes from coded and collated data	Searching codes for clusters of patterned meaning to construct themes. Identify similarity and relationships across the codes	• Immerse in codes to guide initial construction of themes • Consider appropriate theories to achieve the best theoretical interpretation
Phase 4	Developing and reviewing themes	Reviewing the initial themes which led to identification of Person- centred Care, Complex Adaptive Systems and Integrated Care as central concepts	• Interpretation in relation to theories about Person-centred Care, Complex Adaptive Systems and Integrated Care
Phase 5	Refining, defining and naming themes	Deriving one main theme and three sub-themes	

^*^ The phases refer to Braun and Clarke’s thematic analysis[38]

The software programme NVivo, version from March 2020 [42], was used to facilitate coding and collating data.

Based on the systematic data coding process (Phase 2) and generation of initial themes from coded and collated data (Phase 3), the theories about Person-centred Care (PCC) [43–47], Complex Adaptive Systems (CAS) [48–50] and Integrated Care [51–53] were found appropriate to enrich the interpretation of data and create the final themes (Phase 4).

### Person-centred care as theoretical framework for the interpretation

The PCC concept involves an approach where you put the person before the disease. This means focusing on the individual’s needs, preferences and values, rather than favouring the preferences of the healthcare system and the professionals [3, 43–47, 54].

PCC stresses that providers must welcome an individual, respectful, and holistic approach when meeting healthcare users [43, 47, 55]. This highlights the importance of getting to know the person through establishing a good relationship between provider and user [45].

### Complex adaptive systems as a theoretical framework for the interpretation

Within a CAS approach, rehabilitation pathways are regarded as non-linear pathways. Episodes may occur spontaneously and changes in one part of the system often lead to unpredictable changes in other parts of the system [49].

Self-organising interactions occur between the agents within the system and are not controlled externally or hierarchically [50]. Thus, the professionals’ actions are seen as creative and flexible. On the other hand, the agents (the participants and the professionals) can act according to simple rules [49]. This means that agents tend to act on the basis of experience from previous and similar situations, and great emphasis is put on culture. Acting in line with simple rules makes agents less flexible and creative. Co-evolution means the system’s ability to change from within, but is paradoxically also susceptible to external influences, because the agents reflexively adapt their behaviour in relation to external input [48, 49].

In addition, there are several parallel systems, just as there are subsystems within the individual systems. An example is that the professionals act in two systems simultaneously, e.g., partly as a colleague and partly as a health professional. Each system is internally connected and can mutually influence each other [50].

### Integrated care as a theoretical framework for interpretation

We understand integrated care as: “an approach to overcome care fragmentation, especially where this is leading to an adverse impact on people’s care experiences and care outcomes” [31]. Coordination and communication between professionals in health and social care are important components of PCC [44], and a PCC approach is the heart of integrated care [52, 53].

### Ethical considerations

The study was carried out in accordance with the Declaration of Helsinki [56]. Written informed consent to participate in the interviews was obtained before the participants were included. To protect the identity of the participants, they are named by participant 1, participant 2 and so on instead of their real names and exact age and sensitive health-related information are omitted in findings.

In accordance with Danish legislation, formal ethical approval was not required since no biomedical material was included [57]. The study was registered by the Danish Data Protection Agency (Journal no.: 2015-57-0008 and later Journal no.: 2018-529-0001).

Data were stored and analysed in OPEN Analyse, a safe environment that complies with the European General Data Protection Regulation and Danish law for data protection which complies with current national and European data protection regulations [58].

### Findings

In all, 11 participants with IA were included. Please, see Table 3 for characteristics of the included participants.

**Table 3:** Characteristics of participants

Participants	Sex	Age Cate-gory (years)	Family	Disease duration (years)	Work status	Level of education^b^
P 1	F	60-69	Living with a partner, adult children	More than 20	Retired	Basic school
P 2	F	80-85	Living with a partner, adult children	More than 20	Retired	Short-cycle higher education
P 3	F	50-59	Living with a partner, adult children	11-20	Flex job scheme ^a^	Short-cycle Higher education
P 4	M	40-49	Living with a partner, five children living at home	3-10	Full time/ service industry employee	Basic school
P 5	F	50-59	Single, adult children	0-2	Sick leave/ health- and social care employee	Short-cycle higher education
P 6	F	70-79	Single, no children	11-20	Retired	Short-cycle higher education
P 7	F	30-39	Single, one child living at home	0-2	Part-time sick leave/ office employee	Medium-cycle higher education
P 8	F	50-59	Single, adult children	More than 20	Early retirement	Short-cycle higher education
P 9	M	50-59	Living with a partner, adult children	0-2	Full time/ technical employee	skilled worker
P 10	M	70-79	Living with a partner, adult children	More than 20	Retired	Long-cycle higher education
P 11	M	40-49	Single, no children	11-20	Long term sick leave/ service industry employee	Basic school

*P* participant, *F* female, *M* male

^a^ Flex job scheme is a job with special conditions, which considers the person's reduction in capacity to work, due to the disease.

^b^ Level of education: Basic school: 10 years; Short-cycle higher education: 1-2 years; Medium-cycle higher education: 3-4 years; Long-cycle higher education:5-7 years

There was one overarching theme: The importance of a person-centred approach, and three sub-themes: 1) Experiences of empowerment and dis-empowerment, 2) Experiences of communication and coordination and 3) Facing everyday life after discharge. In the following, we describe each of the themes in more detail.

The interpretation is based on data across all the interviews from all the participants. However, the overarching theme, and sub-themes 1 and 2 are primarily presented as stories to illuminate how participants’ experiences and actions changed over time. To document a comprehensive examination of all data and to show diversity in experiences, these stories are supplied with quotes from different participants.

Since sub-theme 3 deals with the time after discharge and thus no attention is paid to experiences and actions over time, this sub-theme is depicted without a story but is illustrated with selected quotes.

### The importance of a person-centred approach

To experience coherence in the rehabilitation, it was important that the participants experienced to meet professionals who listened empathetically, were familiar with the participant’s rehabilitation pathways and acknowledged the participant’s own views rather than focusing on external factors such as guidelines, legislation and standards. Such an approach is in line with PCC [47] and this made the participants feel comfortable and reduced their own work to achieve coherent rehabilitation.

Participant 11 was an example of a participant who had met professionals with and without PCC approaches. He had experienced challenges due to back pain since he was quite young. He felt well-treated in relation to his physical disease with relatively good effect of the pharmacological and physiotherapeutic treatment he received. On the other hand, he expressed dissatisfaction with the job-related rehabilitation in the municipality. He had experienced that a variety of social workers had a lack of understanding of his specific problems regarding his disease and the impact the disease had for his ability to perform his paid work, which hindered active participation in work life and thus coherence in his rehabilitation pathway. He believed that the social workers’ main concern was which of the public purses should finance the cost of his job-related rehabilitation. He told:

Well, they want to take you out of it [a box] as quickly as possible and put you into something else [another box], nowadays it’s called something else than it used to back then. Now it’s ’resource clarification programme’ and ’resource allowance’ and stuff like that (Participant 11).

Subsequently, he had met professionals in the municipality with a more person-centred approach and, he reported on a meeting with a professional who was empathetic, showed understanding and recognition. He said:

There’s no point in just sitting down and expecting things to happen all by themselves. You have to make an effort yourself if you want to get things done... And I think I'm starting to learn that.

The interviewer asked: Okay, so something’s happening in that regard?

He replied: A little is happening, yes……….. Well, since I began coming here, I think I’ve undergone a big change on a personal level, too. Also compared to many years ago.

The interviewer asked what caused the development and he replied: I suppose it’s a mixture of it all, the right people and maybe me ... (Participant 11).

Another participant reported on a person-centred approach, where the staff met her special needs for custom-made shoes and furthermore had an eye for the participant’s special needs to get them granted earlier than she was entitled to.

They do hand them [shoes] out to me shortly before sometimes, because I'm so active and all that, I'm not just an 80-year-old bat sitting in a corner and who doesn’t need anything. I mean, I’m going to wear holes in them anyway. And when you wear the same ones every single day, they do take a lot of wear and tear. But in those cases, they give me a new pair. It’s never been a problem (Participant 8).

Furthermore, Participant 8 later expressed satisfaction that it was the podiatrist who managed to get the shoes approved for her, which meant that she did not have to apply for them herself.

The quotation illustrates how some participants met professionals who acknowledged them as unique individuals with various needs and wishes for their lives with the disease, and this corresponds with the principles of PCC [3, 44–47].

On an organisational level, the two professionals provided horizontal integrated care [59] as they were employed in two different organisations in primary sector. The horizontally integrated care facilitated coherence in this healthcare user’s rehabilitation pathway.

The above-mentioned quotation may also illustrate an example of self-organisation [49], meaning that the professionals, as agents in CAS, act self-organised where interactions with professionals are characterised by flexibility and creativity instead of being ruled by external factors. This implies that in some cases, the professionals stretched their efforts and went to the limit of their powers to accommodate the wishes and needs of the participants.

When the participants met professionals who adhered strictly to guidelines, legislation, and standards, some experienced it as a lack of acknowledgement of them as unique individuals. In these cases, the interactions can be understood as internalised simple rules [49], meaning that the professionals’ reactions were based on experiences from previous and similar situations. In these cases, legislations, habits, and culture shaped the professionals’ actions rather than a PCC approach and this may hinder coherence in the healthcare users’ rehabilitation pathways.

### Experiences of empowerment and dis-empowerment

Some of the participants expressed that they had an overall feeling of control of and were able to influence their rehabilitation process themselves. These are core issues in the concept of empowerment [43, 44, 55]. They saw themselves as strong individuals and did not want their relatives to become involved in decisions regarding care and rehabilitation. They sought out possibilities themselves or had prior knowledge about their options in the healthcare system, opportunities for financial compensation in relation to lack of participation in the labour market and possibilities for social support options such as assistive devices and assistance with personal care. Some were empowered to adjust the provided interventions to match their own needs. This could be a referral to free physiotherapy where they brought the descriptions of the exercises to a local gym, because it gave them a better opportunity to organise the training times and duration themselves.

Participant 2 was predominantly satisfied with the help she had received from the municipality because her needs were met in terms of becoming more self-sufficient and making everyday life work. She had been granted a lift to the 2nd floor, had changes made to her kitchen, assistant devices installed in the bathroom, a walker, a wheelchair and a car that was customised to alleviate her impairments. The applications to the municipality were largely approved without major problems, except for the customised car, where she ended up having to contact both the social worker, the social manager in the municipality and a city council member to put pressure on them.

To create coherence in her pathway she stated that she had used the opportunity to get an explanation or follow-up on information from her electronic medical record by contacting her GP via E-contact.

The recent admission was a good experience for her, but in the last interview, 4-6 months after discharge, she expressed disappointment that the discharge interview and the subsequent outpatient consultation were conducted by physicians she had not previously met. She showed empowerment by contacting her regular rheumatologist who had also referred her to the rehabilitation stay and was promised that the subsequent consultation should take place with the rheumatologist in question.

When the interviewer asked about an experience which she considered positive, she replied:

I don’t want to big myself up, but I think if you can’t, that is, plead your cause, you’re going to have a hard time ... I’ve been able to, you know, join part in the conversation on a sound basis, and then, at the same time, I have received a response ... so the conversation is, sort of, on equal terms (Participant 2).

Contrary to this, some of the participants experienced a lack of energy and power to articulate their wishes and needs and thus felt “small’ and meaningless and tended to give up, they felt humiliated and felt disempowered.

During the three interviews, Participant 3, expressed feelings of disempowerment several times concerning various situations. One situation related to the time upon being discharged from the rehabilitation stay, where she had to contact the municipality to apply for assistive devices to alleviate everyday problems. She told:

You have to get in touch with the Citizen Service Centre yourself and all that, you know? Then someone will make a house call, and then they have to check this and that out, and then they have to do a search ... it’s a lost cause. I can’t be bothered. I simply can’t! (Participant 3).

The disempowered participants could also be seen as passive recipients of services [44–46]. An interpretation could be that they did not meet professionals who encouraged and supported empowerment through a PCC approach [43, 44, 55]. As explanations for feeling disempowered, the participants both reported a negative reputation of the general healthcare and social systems and their own unpleasant experiences with professionals, as mentioned above.

### Experiences of communication and coordination

Experiences of lack of coordination and communication, both between the professionals across hospitals and across different hospitals, municipalities and GPs, were dominant and caused that the healthcare users did not achieve coherence in their rehabilitation pathways. However, this is contrary to PCC, which emphasises the importance of placing the responsibility for coordination in transitions between different departments and sectors [46].

The lack of coordination and communication reflects paucity of both horizontal and vertical integration in an integrated care approach [31]. Lack of coordination and communication between professionals within the same department and between departments in same organisational level in an organisation illustrates paucity of horizontal integration, whereas lack of coordination and communication between professionals in different sectors reflects lack of vertical integration e.g. professionals between primary and secondary sector [31, 59].

The same interpretation can be applied to the problematic electronic communication between the various systems. The participants talked about referrals or discharge letters that had been sent or were promised to be sent by their GP or to hospital departments, but which apparently were not always received.

The lack of coordination across hospitals and sectors can also be interpreted as the individual hospitals, municipalities and GPs belonged to parallel systems, which can complicate communications and coordination across these providers [50]. An example was Participant 7. She was referred to specialised rehabilitation by her GP (belonging to one system) to a specialist ward in a hospital (belonging to another system), and she herself had to reach out to her GP to find out that the referral had been rejected. Furthermore, she was asked to provide the results of a MRI scan and tissue tests herself, as they were not automatically shared between the two institutions involved. While hospitalised for specialised rehabilitation, she had to ask relatives to act as couriers. She unsuccessfully tried to obtain the test results from the tissue samples. Due to lack of communication between these various professionals she had to be re-examined and repeat her medical history. She felt frustrated and said:

You’ve got seven different doctors, each doing their own thing. ... So, I can’t get to talk to the same doctor, even at an investigation unit. How the hell do they expect to be able to find out what’s wrong with me, then? Because they ask me the same questions. (Participant 7).

She reflected on her coordinating role and the subsequent risk:

Because I need to be able to coordinate these things, I might end up accidentally passing on some misinformation to the various professionals (Participant 7).

The example above illustrates that some of the participants felt that they came to work overtime being forced to take on a job, which they were not comfortable with. They did not know the professional language and did not always understand the meaning of the information they passed on and they were not always able to solve the task.

Some participants wanted help to ease their work with communication and coordination tasks and wanted the professionals to take over the efforts. One of the participants put it this way:

So, I would like the occupational therapist to contact my workplace and say, “the chair she is sitting on is wrong for her, we recommend this one” and then my employer will submit an application to the municipality instead for me having to apply for it from the municipality. Because I never get around to it (Participant 3).

Some participants asked for a coordinating person with in-depth knowledge of the content in their medical record, was familiar with their preferences, had knowledge about possible available treatment and support and who thus, in collaboration with them, could help them make the most of their rehabilitation efforts.

Participants who experienced that staff did coordinate and communicate across departments felt they were relieved of a heavy burden on their shoulders. A participant experienced that the professionals mutually informed each other. He told:

Well, they knew 100% what was going on, so it's very positive (Participant 4).

The participants who reported on professionals who were a bit more relaxed in terms of guidelines and professional boundaries, experienced being well-helped to experience coherent rehabilitation. In these cases, the professionals had an eye for the participants’ individual wishes, needs, resources and values, cf. PCC [3].

Cases where the professionals stretched their efforts in relation to guidelines and subject boundaries, can be interpreted as self-organisation [50], i.e. they adapted their actions to the participants’ wishes and needs.

### Facing everyday life after discharge

During the admission to specialized rehabilitation, the professionals took some initiatives for the participants to be able to maintain or improve their level of function or make it possible to actively participate in social life after discharge. Although apparently intended as help to achieve coherence in the participants’ rehabilitation pathways, the participants did not always find them helpful. An example was free physiotherapy (physiotherapy is free of charge for patients diagnosed with rheumatoid arthritis in Denmark), because some of the participants apparently did not understand the way the physiotherapy was provided, i.e. on a fixed weekday and time. They wanted, for example, to get started on or maintain physical exercise, but the free physiotherapy often took place under circumstances that didn’t match their needs and would lead to new restrictions in their everyday lives, e.g., if the time was incompatible with their work.

In CAS, agents’ actions are influenced by interactions and are thus performed in a non-linear way [49, 50]. This conflicts with the idea that our actions are predominantly expressions of rational and goal-oriented decisions [60]. A contributing interpretation is that the participants initially agreed on the professionals’ initiatives; however, it turned out to be difficult for them to comply with this after discharge, because the participants had to make their everyday lives work in completely different contexts, which meant they were involved in other social interactions, in other cultures and systems than experienced during their rehabilitation stay.

The fact that it can be easier to perform exercise during hospitalisation as they have more time to exercise than at home may also be attributed to the importance of natural co-evolution [48, 49]. This means that the participants managed to acquire the necessary skills and actions to be able to perform in the best possible way during their stay, such as to live up to the role as a patient by training as agreed with the professionals – i.e., the participants changed their actions and internalised these in their systems, at least during hospitalisation.

One of the participants had been admitted several times for specialised rehabilitation and each time she experienced new energy also in relation to wanting to continue training after discharge. She was a wheelchair user but could walk around a bit and she found herself to achieve better balance, become more confident and better able to move around during her recent hospitalisation. In the first interview after the admission, she talked about the boost she experienced immediately after the admission and which she also knew from the previous times. In her words:

As always, when I’ve been there, I feel really positively energised. If your outlook on life has been even slightly bleak, it goes away. They are full of goodness, but they also give you a shot in the arm around the clock (Participant 6).

## Discussion

The findings documented that the participants’ rehabilitation work was facilitated when professionals met them in accordance with the principles of PCC [3, 44–47]. The participants experienced coherence in their rehabilitation when the professionals saw them as unique individuals with their own values and norms. Previous research is consistent with this regarding increased satisfaction and well-being when the patients experience PCC [43, 61, 62]. Patients with chronic diseases wish to be treated “as a whole, not as a series of separate problems” [63].

Furthermore, our study documented that the healthcare users experienced lack of coordination and communication between the various professionals employed across diverse departments, organisations and sectors, which is in line with previous studies [14, 64]. Both CAS and Integrated Care complemented the PCC approach in the interpretation of data. CAS added an understanding of the complexity of the health care users’ rehabilitation pathways and the organisational issues, including communication, collaboration and the organisational challenges in achieving coherence for the healthcare users [48, 49, 65]. Integrated Care supplemented with an organisational understanding of the significance of both horizontal and vertical integration [31, 59].

The participants experienced lack of coordination and communication as a barrier for a coherent rehabilitation pathway. To achieve coherence in the fragmented services, the participants felt compelled to manage the coordination themselves and they often felt they lacked the necessary knowledge and competences and thus felt frustrated and that they were working overtime. These findings are supported by previous research of rehabilitation processes in Norway [5].

Other studies have also paid attention to the workload of people living with chronic conditions under the concept of treatment burden [66–70]. Treatment burden refers to the workload people living with a chronic condition are expected to adhere to and is thus related to self-management. Our participants primarily felt overloaded, when they met professionals, who did not use a PCC approach and when they experienced lack of coordination and communication. Participant 11 was an example who suffered from treatment burden as he was not meet with a PCC approach in job-related rehabilitation relation. Participant 7 had to organise that the results from MRI test and tissue test arrived from one hospital to another. Participant 8 felt relieved from the treatment burden, because she was met with a PCC approach and got her custom-made shoes, and it was the podiatrist who managed to get the shoes approved for her.

It is already documented that the healthcare system can contribute to treatment burden through poor care coordination, meeting professionals, who do not use a person-centred approach, and inadequate information [68, 70]. Thus, the professionals must be aware of the risk to pass treatment burden on to healthcare users.

Furthermore, our results call for effective electronic systems to share information between professionals, particularly across organisational boundaries. This is also supported by a previous British study [14]. The British study was based on interviews with patients, carers and professionals and investigated how the organisation of health and social care could reduce older people’s use of emergency hospital [14].

Our study showed that when professionals deviate a little from guidelines and professional boundaries, it helps facilitate coherence in the participants’ rehabilitation pathway i.e., the podiatrist who granted Participant 8 a pair of custom-made shoes earlier than she was entitled to according to local guidelines.

The complexity of the participants’ needs and the professionals’ agility to make changes accordingly made it possible for the participants to experience coherence. This is acknowledged both within integrated care and CAS [71]. Previous studies indicate that a mindset where integrated care is combined with the characteristics of CAS can probably help to overcome problems with coordination and communication [71–74].

However, it can be problematic if fragmentation is solved only locally by the single professional because the efforts thus become dependent on the individual professionals’ creative solutions. Therefore, it is necessary that such initiatives are supported from the managerial level within and across organisations. This requires that policy makers and organisations have the capacity to handle care and rehabilitation pathways characterised by complexity to collaborate together to achieve coherent rehabilitation pathways [73].

The PCC approach was experienced positively by the participants in this study, and politically, legislatively and locally in the healthcare system, it is encouraged that the healthcare system is supported by this approach [75, 76]. At the same time, Dahlborg and colleges argue that the healthcare system should be carried by a set of values in line with the new public management (NPM) principles [76]. The study regarding NPM is relevant to a Danish context as Denmark’s public sector has undergone comprehensive reforms over the past decades. The principles from NPM have influenced these reforms and still play a significant role in health and social care [77, 78].

Thus, there seems to be two conflicting discourses in the healthcare system. In line with the PCC mindset, healthcare users are considered active and self-determining people. The second discourse on NPM reflects a demand for high productivity and efficiency in the healthcare system, where professionals, having expert knowledge, seem best equipped to assess the most appropriate decision seen solely from a diagnostic and treatment perspective in relation to disease [76]. These two conflicting discourses can cause dilemmas for professionals in relation to who, what and which values they must consider when discussing and deciding on which measures and actions to implement in relation to the healthcare users [79].

Finally, professionals in the secondary sector i.e, healthcare professionals employed in hospitals, focus on diagnoses and test results. This can be part of a hospital culture with a biomedical focus rather than a rehabilitation culture with a focus on disabilities and bio-psycho-social functioning. A PCC approach must be prioritised over a NPM approach if the general health- and social care aim for coherence for the patients.

To address the lack of coordination, some of the participants called for a coordinating person. Patient navigators are often used in terms of integrated care, especially for individuals with complex care needs [80–83]. Even though positive outcomes of patient navigators are difficult to measure due to unclear definitions of the concept, and what and from whose perspective the outcomes should be measured [84], patient navigators may help to facilitate coordination tasks by supporting and guiding patients through the system. Patients who have been offered contact with patient navigators have reported increased access to care, improvements in health and wellness, increased satisfaction, improved self-efficacy, self-management, and empowerment [85].

### Strengths and limitations

We find that the concept information power strengthened the study as a concept to achieve internal validity [36]. We evaluated the achievement of information power regarding study aim, sample specificity, quality of dialogue, use of analysis strategy and established theory [36]. Regarding study aim and sample specificity including maximum variation [38], we succeeded in recruiting participants with variations in age and duration of disease to answer the study aim. Due to variation in sex, we had to pause in including females and thereby we succeeded in balancing the number of males and females.

We find that we established a positive and confident relation and thereby achieved good dialogues with the participants during the three interviews with each participant. Along with the researchers’ knowledge about the participants and the rehabilitation pathways, it was easy to determine whether the participants felt comfortable to express their views during the dialogue. We thus consider it a strength that the participants were followed for a relatively long time, i.e., 6-8 months, which also made it possible to observe and hear the participants’ views temporally close to their experiences, but also to follow how their experiences and actions changed over time.

Information power is claimed to fit with Braun and Clark’s TA analysis strategy and its underlying epistemology, because they adhere to the interpretive part of the continuum in qualitative research instead of the descriptive and neopositivist part [86]. The initial analysis with the open coding followed by the use of established theory increased information power and thus we consider it a strength that the data were interpreted based on CAS, PCC and integrated care as we find this helped lift the analysis to a higher level of abstraction [87].

Another strength is the diversity in the competences of the team behind this study. This included the patient research partners contributions in the interpretation of the findings as they could relate to their own experiences.

A limitation of the study is that the participants were recruited from only one specific hospital in connection with their admission to specialised rehabilitation, which may imply that the findings tend to reflect local conditions. However, the participants are residents from several parts of Denmark, and thus their experiences reflect contacts with the healthcare services and local authorities from several locations in Denmark. Furthermore, several of our findings are supported by the findings from other studies.

Another limitation is, that some of the participants might have had special interests driving their desire to participate in the current study. Some might think they were able to benefit from better therapeutic efforts, closer monitoring, and access to new treatments, wanting to help other patients or a more general altruism [88]. Some participants do not want to participate in research due to their poor health status [88].

We did not aim to achieve coding agreement between different members of the team in the initial coding in accordance with Reflexive TA [40] as we acknowledge that the analysis may lead to variations in interpretations depending on the researcher’s professional background, knowledge and understanding [40]. Some might consider this approach as a limitation.

Although we only included 11 participants, the relatively small number provided excellent opportunities to carry out in depth interviews more times with each participant, which provided understanding for barriers and facilitators to achieve coherence over time.

This study solely focused on the healthcare users’ and not the professionals’ perspective. It is relevant also to examine how the professionals view their role in the rehabilitation pathways and how the organisational and cultural frameworks affect both the professionals’ and healthcare users’ opportunities to create coherent rehabilitation.

## Conclusion

Coherent rehabilitation pathways may be facilitated when healthcare users in rehabilitation pathways meet professionals with a person-centred approach. This means to meet healthcare users with an individual, respectful, and holistic approach who meet their individual needs.

Meeting professionals who do use not use a person-centred approach causes barriers to achieve coherence in the rehabilitation pathway. Lack of communication and coordination between the participants in rehabilitation and professionals, and between the different professionals within and across departments and sectors lead to experiences of fragmentations in their pathways.

## Data Availability

The datasets generated and/or analysed during the current study are not publicly available due to Danish law but are available from the corresponding author on reasonable request.

## Abbreviations

IA: Inflammatory Arthritis
GP: General Practitioner
TA: Thematic reflexive analysis
PCC: Person-centred Care
CAS: Complex Adaptive Systems
NPM: New public management

## References

[1] Gröne O, Garcia-Barbero M. Integrated care: a position paper of the WHO European office for integrated health care services. Int J Integ Care. 2001;1. Available from: https://www.ncbi.nlm.nih.gov/pmc/articles/PMC1525335/ [09 Nov 2022] PMC1525335 16896400

[2] Buch MS, Jensen MCF, Brorholt G. Sammenhæng i patientforløb – Hvilke modeller og anbefalinger er der, og hvordan kan de bruges i praksis? [Coherence in patient pathways - What models and recommendations are there and how can they be used in practice?]. VIVE – Viden til Velfærd. Det Nationale Forsknings- og Analysecenter for Velfærd [VIVE – Knowledge for Welfare. The National Research and Analysis Center for Welfare] 2018. København. Available from: https://www.vive.dk/media/pure/10770/2302286 [9 Nov 2022]

[3] World Health Organization. WHO global strategy on people-centred and integrated health services: interim report. Geneva: World Health Organization; 2015.

[4] Organization WH. World report on ageing and health. Luxembourg: World Health Organization; 2015.

[5] Breimo JP (2015). Captured by care: an institutional ethnography on the work of being in a rehabilitation process in Norway. J Soc Soc Welfare.

[6] Martin MH. Er der styr på mig [Is there control over me?] Dansk Sygehus institut 2010. Available from: https://www.vive.dk/da/udgivelser/er-der-styr-paa-mig-9335/. [16 Sep 2019].

[7] World Health Organization (2017). Rehabilitation 2030: a call for action Geneva.

[8] World Health Organization. Rehabilitation in health systems - a guide for action Geneva; 2017. Available from: https://www.who.int/publications/i/item/9789241515986.

[9] Søndergaard EG, Grøne BH, Wulff CN (2013). A survey of cancer patients’ unmet information and coordination needs in handovers–a cross-sectional study. BMC Res Notes.

[10] Danske patienter. Mangel på sammenhæng i sundhedsvæsenet - fortalt af patienter og pårørende. [Lack of coherence in healthcare - told by patients and relatives] 2013. Available from: https://danskepatienter.dk/files/media/Publikationer%20-%20Egne/A_Danske%20Patienter/B_Indspil_cases_unders%C3%B8gelser/manglende_sammenhaeng_i_sundhedsvaesenet_web_januar2014.pdf.

[11] Danske patienter. Tættere på trivsel [Closer to Well-being] 2021. Available from: https://danskepatienter.dk/sites/danskepatienter.dk/files/media/document/Unders%C3%B8gelse_T%C3%A6ttere%20pa%CC%8A%20trivsel.pdf.

[12] Schiøtz ML, Høst D, Frølich A (2016). Involving patients with multimorbidity in service planning: perspectives on continuity and care coordination. Journal of comorbidity..

[13] Nolte E, Knai C, Hofmarcher M (2012). Overcoming fragmentation in health care: chronic care in Austria, Germany and The Netherlands. Health Econ Policy Law.

[14] Beech R, Henderson C, Ashby S (2013). Does integrated governance lead to integrated patient care? Findings from the innovation forum. Health Soc Care Community.

[15] Sampson R, Cooper J, Barbour R (2015). Patients’ perspectives on the medical primary–secondary care interface: systematic review and synthesis of qualitative research. BMJ Open.

[16] Wallin M, Talvitie U, Cattan M (2007). The meanings older people give to their rehabilitation experience. Ageing Soc.

[17] Klein J, Hopper T (2013). Restructuring a rehabilitation program for older adults: effects on patient outcomes and staff perspectives. Can J Aging.

[18] Graff HJ, Christensen U, Poulsen I (2018). Patient perspectives on navigating the field of traumatic brain injury rehabilitation: a qualitative thematic analysis. Disabil Rehabil.

[19] Miettinen S, Ashorn U, Lehto J. Talking about the institutional complexity of the integrated rehabilitation system—the importance of coordination. Int J Integrated care. 2013;13. Available from: https://www.ncbi.nlm.nih.gov/pmc/articles/PMC3653285/ [09 Nov 2022] 10.5334/ijic.851 PMC3653285 23687479

[20] Hope. European Hospital and Healthcare Federation. Health professionals in Europe: new roles, new skills. 2009. Available from: https://hope.be/wp-content/uploads/2016/02/HOPE-exchange_2009-synthesis.pdf. [09 Nov 2022]

[21] European Commision. Task shifting and health system design. Luxemburg 2019. Available from: https://health.ec.europa.eu/system/files/2019-11/023_taskshifting_en_0.pdf. [09 Nov 2022]

[22] Doessing A, Burau V (2015). Care coordination of multimorbidity: a scoping study. J Comorbidity.

[23] Johansen J, Rahbek J, Møller K, et al. Rehabilitering i Danmark, Hvidbog om rehabiliteringsbegrebet [Rehabilitation in Denmark, White paper of the rehabilitation concept]. Aarhus: MarselisborgCentret; 2004. Available from: https://www.rehabiliteringsforum.dk/contentassets/519c219e42a3454da34db60a8454de58/hvidbog.pdf. [09 Nov 2022]

[24] OECD. OECD Reviews of Health Care Quality: Denmark 2013 2013 Available from: https://www.oecd.org/els/health-systems/ReviewofHealthCareQualityDENMARK_ExecutiveSummary.pdf. [27 Sep 2021].

[25] Schmidt M, Schmidt SAJ, Adelborg K (2019). The Danish health care system and epidemiological research: from health care contacts to database records. Clin Epidemiol.

[26] Lenssinck ML, Burdorf A, Boonen A (2013). Consequences of inflammatory arthritis for workplace productivity loss and sick leave: a systematic review. Ann Rheum Dis.

[27] Hansen SM, Hetland ML, Pedersen J (2016). Effect of rheumatoid arthritis on longterm sickness absence in 1994–2011: a Danish cohort study. J Rheumatol.

[28] Feddersen H, Mechlenborg Kristiansen T, Tanggaard Andersen P (2018). Juggling identities of rheumatoid arthritis, motherhood and paid work - a grounded theory study. Disabil Rehabil.

[29] Dagfinrud H, Kjeken I, Mowinckel P (2005). Impact of functional impairment in ankylosing spondylitis: impairment, activity limitation, and participation restrictions. J Rheumatol.

[30] Kristiansen TM, Primdahl J, Antoft R (2012). Everyday life with rheumatoid arthritis and implications for patient education and clinical practice: a focus group study. Musculoskeletal Care.

[31] Understanding GN, Care I (2016). Understanding Integrated Care. Int J Integr Care.

[32] Gonzalez-Ortiz LG, Calciolari S, Goodwin N (2018). The core dimensions of integrated care: a literature review to support the development of a comprehensive framework for implementing integrated Care. Int J Integr Care.

[33] Tong A, Sainsbury P, Craig J (2007). Consolidated criteria for reporting qualitative research (COREQ): a 32-item checklist for interviews and focus groups. Int J Qual Health Care.

[34] Blumer H. Symbolic interactionism: Perspective and method. CA: Univ of California Press; 1969/1986.

[35] Holstein JA, Gubrium J, Silverman D (2004). The active interview. Qualitative Research: Theory, Method and Practice.

[36] Malterud K, Siersma VD, Guassora AD (2016). Sample size in qualitative interview studies: guided by information power. Qual Health Res.

[37] Sim J, Saunders B, Waterfield J (2018). Can sample size in qualitative research be determined a priori?. Int J Soc Res Methodol.

[38] Patton MQ (2015). Qualitative research and methods: Integrating theory and practice.

[39] McIntosh MJ, Morse JM (2015). Situating and Constructing Diversity in Semi-Structured Interviews. Glob Qual Nurs Res.

[40] Braun V, Clarke V. One size fits all? What counts as quality practice in (reflexive) thematic analysis? Qual Res Psychol. 2020:1-25.

[41] Braun V, Clarke V, Hayfield N, Liamputtong P (2019). Thematic analysis. Handbook of Research Methods in Health Social Sciences.

[42] QSR international. NVivo 2020. Available from: https://support.qsrinternational.com/s/article/What-is-the-latest-version-of-NVivo

[43] Yun D, Choi J (2019). Person-centered rehabilitation care and outcomes: A systematic literature review. Int J Nurs Stud.

[44] Morgan S, Yoder LH (2012). A concept analysis of person-centered care. J Holist Nurs.

[45] Ekman I, Swedberg K, Taft C (2011). Person-centered care–ready for prime time. Eur J Cardiovasc Nurs.

[46] Collins A. Measuring what really matters.Towards a coherent measurement system to support person-centred care. London2014; 1-20]. Available from: https://www.health.org.uk/sites/default/files/MeasuringWhatReallyMatters.pdf.

[47] McCormack B, Borg M, Cardiff S, et al. Person-centredness-the'state'of the art. Int Pract Dev J. 2015;5(Special Issue). Available from: https://browzine.com/libraries/1182/journals/64715/issues/202018488. [09 Nov 2022]

[48] Martin C, Sturmberg J (2009). Complex adaptive chronic care. J Eval Clin Pract.

[49] Plsek PE, Greenhalgh T (2001). Complexity science: the challenge of complexity in health care. BMJ (Clinical research ed).

[50] Cilliers P. Understanding complex systems. Handbook of systems and complexity in health. New York: Springer; 2013. p. 27-38.

[51] Goodwin N, Stein V, Amelung V, Amelung V, Stein V, Suter E (2021). What is Integrated Care?. Handbook Integrated Care.

[52] Shaw S, Rosen R, Rumbold B (2011). What is integrated care.

[53] Greenfield G, Ignatowicz AM, Belsi A (2014). Wake up, wake up! It's me! It's my life! patient narratives on person-centeredness in the integrated care context: a qualitative study. BMC Health Serv Res.

[54] Jesus TS, Bright FA, Pinho CS (2021). Scoping review of the person-centered literature in adult physical rehabilitation. Disabil Rehabil.

[55] World Health Organization. Regional Office for the Western P. People-centred health care : a policy framework: Manila : WHO Regional Office for the Western Pacific; 2007 [updated 2007; cited 2020 08 06]. Available from: https://apps.who.int/iris/handle/10665/206971

[56] World Medical Association. Declaration of Helsinki 2018. Available from: https://www.wma.net/policies-post/wma-declaration-of-helsinki-ethical-principles-for-medical-research-involving-human-subjects/. [3 Jul 2019]

[57] Sundheds- og Ældreministeriet. LBK nr 1338 af 01/09/2020 2020. Available from: https://www.retsinformation.dk/eli/lta/2020/1338. [Cited 2021 12 April].

[58] Forskningsenheden OPEN RUO. Available from: https://open.rsyd.dk/forskningsenheden-open. [15 08 2022].

[59] Goodwin N (2013). Understanding integrated care: a complex process, a fundamental principle. Int J Integrated Care.

[60] Cohn S (2014). From health behaviours to health practices: an introduction. Sociol Health Illn.

[61] Rathert C, Wyrwich MD, Boren SA (2013). Patient-centered care and outcomes: a systematic review of the literature. Med Care Res Rev.

[62] Bala SV, Forslind K, Fridlund B (2018). Person-centred care in nurse-led outpatient rheumatology clinics: conceptualization and initial development of a measurement instrument. Musculoskeletal Care.

[63] Wade D (2016). Rehabilitation–a new approach. Part four: a new paradigm, and its implications.

[64] Schoen C, Osborn R, Squires D (2011). New 2011 survey of patients with complex care needs in eleven countries finds that care is often poorly coordinated. Health Aff (Millwood).

[65] Sturmberg JP, Cilliers P (2009). Time and the consultation-an argument for a 'certain slowness'. J Eval Clin Pract.

[66] Tran VT, Montori VM, Eton DT (2012). Development and description of measurement properties of an instrument to assess treatment burden among patients with multiple chronic conditions. BMC Med.

[67] Tran VT, Harrington M, Montori VM (2014). Adaptation and validation of the Treatment Burden Questionnaire (TBQ) in English using an internet platform. BMC Med.

[68] Sav A, Whitty JA, McMillan SS (2016). Treatment Burden and Chronic Illness: Who is at Most Risk?. Patient.

[69] Schreiner N, Perazzo J, Currie J (2019). A descriptive, cross-sectional study examining treatment burden in people living with HIV. Appl Nurs Res.

[70] Schreiner N, Daly B (2020). A pilot study exploring treatment burden in a skilled nursing population. Rehabil Nurs.

[71] Edgren L, Barnard K. Complex adaptive systems for management of integrated care. Leadersh Health Serv. 2012;25(1):39-51.

[72] Nugus P, Carroll K, Hewett DG (2010). Integrated care in the emergency department: a complex adaptive systems perspective. Social Sci Med (1982).

[73] Grudniewicz A, Tenbensel T, Evans JM (2018). ‘Complexity-compatible’ policy for integrated care? Lessons from the implementation of Ontario's Health Links. Soc Sci Med.

[74] Tsasis P, Evans JM, Owen S (2012). Reframing the challenges to integrated care: a complex-adaptive systems perspective. Int J Integr Care.

[75] Aasen EM, Crawford P, Dahl BM (2020). Discursive construction of the patient in online clinical cancer pathways information. J Adv Nurs.

[76] Dahlborg E, Tengelin E, Aasen E, et al. The struggle between welfare state models and prevailing healthcare policy in Scandinavian healthcare legislative documents. Intl J Health Gov. 2021;26(1):51-64.

[77] Andrews R, Beynon MJ, McDermott A (2019). Configurations of New Public Management reforms and the efficiency, effectiveness and equity of public healthcare systems: a fuzzy-set qualitative comparative analysis. Public Manag Rev.

[78] Terlizzi A, Esposito G. New Public Management Reform ideas and the remaking of the Italian and Danish health systems. Territory, Polit, Gov. 2021:1-20. Available from: https://www.tandfonline.com/doi/full/10.1080/21622671.2021.1930129. [09 Nov 2022]

[79] Persson MH, Mogensen CB, Søndergaard J (2021). Healthcare professionals’ practice and interactions in older peoples’ cross-sectoral clinical care trajectories when acutely hospitalized-a qualitative observation study. BMC Health Serv Res.

[80] Doucet S, Luke A, Splane J, et al. Patient navigation as an approach to improve the integration of care: the case of NaviCare/SoinsNavi. Int J Integr Care. 2019;19(4):1–6. 10.5334/ijic.4648 PMC6857520 31798358

[81] McMullen L, Oncology nurse navigators and the continuum of cancer care. Seminars in Oncology Nursing; 2013;29(2):105-17. 10.1016/j.soncn.2013.02.005 23651680

[82] Fillion L, Cook S, Veillette A-M, et al., editors. Professional navigation framework: elaboration and validation in a Canadian context. Oncol Nurs Forum. 2012;39(1):58-69. 10.1188/12.ONF.E58-E69 22201669

[83] Reid AE, Doucet S, Luke A (2019). The impact of patient navigation: a scoping review protocol. JBI Database System Rev Implement Rep.

[84] McBrien KA, Ivers N, Barnieh L (2018). Patient navigators for people with chronic disease: a systematic review. PLoS ONE.

[85] Carter N, Valaitis RK, Lam A (2018). Navigation delivery models and roles of navigators in primary care: a scoping literature review. BMC Health Serv Res.

[86] Braun V, Clarke V (2021). To saturate or not to saturate? Questioning data saturation as a useful concept for thematic analysis and sample-size rationales. Qual Res Sport, Exerc Health.

[87] Braun V, Clarke V (2019). Reflecting on reflexive thematic analysis. Qual Res Sport, Exerc Health.

[88] Sheridan R, Martin-Kerry J, Hudson J (2020). Why do patients take part in research? An overview of systematic reviews of psychosocial barriers and facilitators. Trials.

